# Organizing a COVID-19 triage unit: a Swiss perspective

**DOI:** 10.1080/22221751.2020.1787107

**Published:** 2020-07-07

**Authors:** Georgios Peros, Ferda Gronki, Nadine Molitor, Michael Streit, Kiyoshi Sugimoto, Urs Karrer, Fabian Lunger, Michel Adamina, Stefan Breitenstein, Tenzin Lamdark

**Affiliations:** a Department of Surgery, Cantonal Hospital Winterthur, Winterthur, Switzerland; b University Heart Center, University Hospital Zurich, Zurich, Switzerland; c Department of Medicine, Cantonal Hospital Winterthur, Winterthur, Switzerland; d Faculty of Medicine, University of Basel, Basel, Switzerland

**Keywords:** COVID, COVID-19, Sars-CoV-2, triage, Swiss

## Abstract

**Background:** With the rapid global spread of the acute respiratory syndrome coronavirus 2, urgent health-care measures have been implemented. We describe the organizational process in setting up a coronavirus disease 2019 triage unit in a Swiss tertiary care hospital. **Methods:** Our triage unit was set-up outside of the main hospital building and consists of three areas: 1. Pre-triage, 2. Triage, and 3. Triage *plus*. The Pre-triage check-points identify any potential COVID-19-infected patients and re-direct them to the main Triage area where trained medical staff screen which patients undergo diagnostic testing. If testing is indicated, nasopharyngeal swabs are performed. If patients require further investigations, they are referred to Triage *plus*. At this stage, patients are then discharged home after additional testing or admitted to the hospital for management. **Observations:** A total of 1265 patients were screened between 10 March 2020 and 12 April 2020 at our Triage unit. Of these, 112 (8.9%) tested positive. 73 (65%) of the positively-tested patients were female and 39 (35%) were male. The mean age for all patients was 43.8 years (SD 16.3 years). Distinguishing between genders, mean age for females was 41.1 (SD 16.5) and mean age for males was 48.6 (SD 14.9), with females being significantly younger than males (*p* < 0.001). **Conclusion:** Our triage unit was set-up as part of a large-scale restructuring process. Current challenges include low sensitivity for test results as well as limited staff and resources. We hope that our experience will help other health care institutions develop similar triage systems.

## Background

The novel coronavirus pandemic that began in Wuhan, China in December 2019 [1,2], also known as the severe acute respiratory syndrome coronavirus 2 (SARS-CoV-2), has caused a global impact on health, economy, and politics in the last few months. The clinical syndrome from the virus, now termed coronavirus disease 2019 (COVID-19), can range from mild respiratory symptoms and fever in mild cases, to acute respiratory distress syndrome (ARDS) and death in severe cases [2,3]. The Swiss Federal Council declared the rapid world-wide spread of COVID-19 infection an “extraordinary situation” [4,5] and on 11 March 2020 the viral spread was officially declared a pandemic by the World Health Organisation (WHO) [1].

Switzerland currently has one of the highest incidence of COVID-19 [6,7] worldwide among affected countries, but also one of the lowest mortalities, with a case fatality ratio of approximately 0.054 [8] (number of fatalities per reported cases). On 23 April 2020, the John Hopkins University coronavirus resource centre reported 28,268 confirmed COVID-19 cases and 1509 deaths [9] in Switzerland, which has a population of 8,603,900 residents [10]. Hence, Switzerland ranks 15th in absolute numbers of confirmed cases despite of a nationwide policy of screening only symptomatic individuals. On 16 March 2020, the Swiss government introduced drastic new social-distancing measures to prevent further dissemination of the virus and to protect the health and safety of the public [5]. Hospital infrastructures across Switzerland were re-organised to cope with the anticipated influx of critically ill COVID-19 patients, including a significant upscale of the intensive care unit and mechanical ventilation [11].

In preparation for the pandemic at a local level, our hospital, a 500-bed tertiary care centre serving a population of about 500,000 local residents, underwent major re-organization at both management and procedural levels. A crisis management team or hospital task force, including the hospital chief executive officer, was formed. The task force met on a daily basis and oversaw significant overhaul to the hospital’s infrastructure. First, an external triage unit for screening and managing potential COVID-19 patients was set up. Second, all elective hospital procedures were suspended. Third, the intensive care unit (ICU) capacity was increased from 14 to 44 beds, including the conversion of five operating rooms into 20 fully-equipped ICU beds. While all three elements are critical in preparation for the pandemic, this article focuses on the rationale and organizational process behind our COVID-19 triage unit set-up.

## Rationale

**Figure 1.**
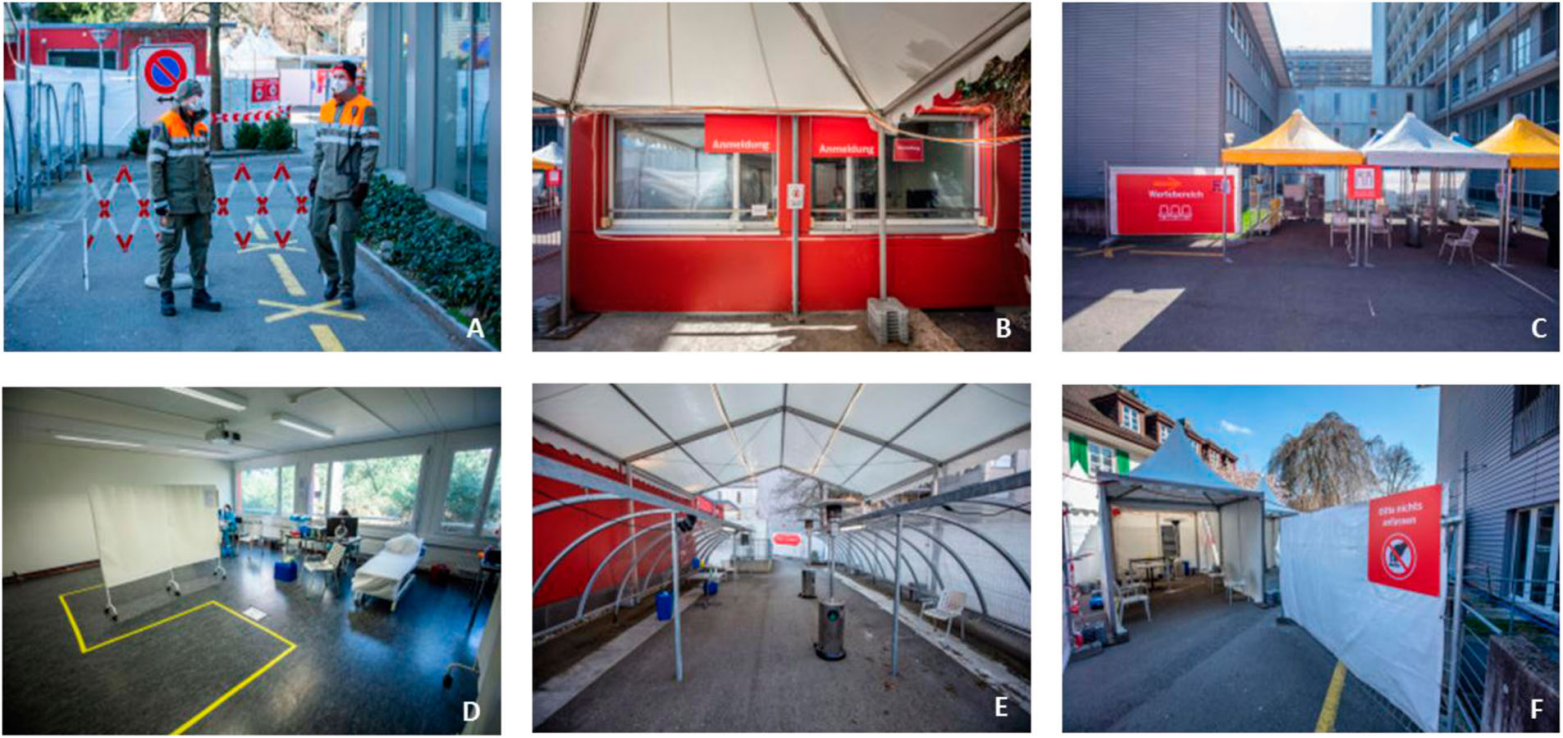
**(A–E):** Triage Unit set-up. **A**: Pre-Triage check point operated by Military staff. **B**: Patient reception, **C**: Waiting area, **D**: Clinical assessment room, **E**: Testing area for nasopharyngeal specimen collection, **F**: Triage *plus* tent.

Critical control of COVID-19 relies on its prompt early identification, appropriate risk evaluation, isolation of possible cases and prevention measures for the spread of the virus [12]. A vital strategy in minimising the risk of infection begins with an effective triage system. Our triage unit was strategically constructed outside the main hospital building to help prevent the spread of COVID-19 through early screening, before potentially affected individuals even enter the hospital premise. Patients who are identified as meeting COVID-19 criteria or who have tested positive for the disease are categorized as suspected or confirmed cases, respectively, and are immediately re-directed into designated isolation zones. Triaging outside the hospital premise has the additional strategic function of distinguishing between patients with mild to moderate symptoms and no risk factors, who are eligible for outpatient care and may require home isolation only, and those with severe illness and/or additional risk factors, who require further investigation or hospitalization [4,13]. The concept consists of three areas: 1. Pre-triage, 2. Triage and, 3. Triage *plus* (Figure 1).

## Methods

### Pre-triage

The aim of pre-triaging is to allow a select group of individuals, including patients who do not fulfil COVID-19 criteria and all hospital staff, to enter the hospital building. All hospital staff who enter the premise for work have to present a hospital identification in order to gain access to the hospital facilities. The pre-triage areas or check-points are located in front of each of the two main entrances to the hospital. These check-points are not operated by medical staff, but rather by the hospital security, as well as the Civil Protection Unit, a division of the Swiss Military Defence [14], which protects the population and its vital resources in the event of disasters, major emergencies and armed conflict. The personal protective equipment (PPE) at this stage include surgical masks and goggles [15]. Hand sanitizer (80% Ethanol, 1% Glycerin) and gloves are also provided. No patient contact is allowed and a minimal distance of 2 metres (≈3.28 feet) is maintained at all times.

Individuals without COVID-19 symptoms who have an outpatient clinic appointment are allowed to enter the main hospital building only after presenting their clinic appointment letter. Patients admitted to the Emergency Department (ED), but who are otherwise clinically stable, enter the ED via the pre-triaging process. Unstable critically ill patients enter the ED. In these cases, family members and visitors are generally not allowed to enter the hospital, except in special circumstances.

Patients with COVID-19 symptoms are immediately provided surgical masks and are asked to proceed to the main Triage area using a separate route. Accompanying friends or family members are instructed to maintain a minimal distance of 2 metres (≈3.28 feet) distance, unless this is not possible, such as in the case of small children or elderly patients requiring assistance.

### Triage

#### Structure

The main Triage area consists of one pavilion measuring approximately 120 square metres (≈1290 square feet) and three large outdoor tents located adjacent to the main hospital building. Tents were chosen instead of containers for better ventilation and wider space capacity, mitigating close contact among individuals. Inside the pavilion, internal doors were removed to allow free access and two bathroom facilities were sealed off to minimize contamination. The remaining five rooms were modified as follows: one is used as a patient reception and administration area, two as rooms for patient assessment, one as a storage unit for PPE and hand sanitizers, and one as a conference room for small staff meetings. The floors are clearly marked with arrows to show the patients’ route.

The three large outdoor tents are used for: the patient reception area, the waiting area, and the testing area for nasopharyngeal specimen collection. These tents have open sides to further increase ventilation. Areas designation and rules for patients are clearly written in white lettering on large red signs.

By the patient reception area, there is also a separate building with dedicated facilities for the triage staff such as a dining area, dressing room and toilettes. No recently-used PPE is permitted in this building, and no more than five individuals can be present in these communal areas at any given time. The COVID-19 triage employees work as a separate unit from the main hospital staff and remain physically separated from the main hospital building to minimize the risk of virus transmission between health care workers (HCW). In fact, there is a 48-hour self-isolation period before triage employees are allowed to re-enter the main hospital.

The hours of operation at the Triage area are between 8am and 10pm, with staff rotating between 2 shifts. After 10pm, triaging for COVID-19 patients is performed in a designated zone in the ED.

#### Staff & equipment

The PPE for all staff, except the physicians, are surgical masks and goggles [15], with hand sanitizer (80% Ethanol, 1% Glycerin) and gloves also available. Patients are provided a chair to sit. This chair is then cleaned and disinfected after every use. Physicians who must come closer than 2 m (≈3.28 feet) to patients in order to perform the nasopharyngeal swabs, are equipped with filtering face pieces with protection 2 (FFP2) respirators, long sleeve fluid repellent gowns, polycarbonate safety glasses and disposable gloves. These physicians, who are responsible for directly screening suspected COVID-19 patients and performing the swabs, are careful not to make any direct contact with the patients themselves or with other staff members. If direct physical contact with patients has been inadvertently made, then the contaminated gowns and gloves are disposed of in clearly labelled bins and fresh protective gear is then re-applied.

#### Procedure

**Figure 2.**
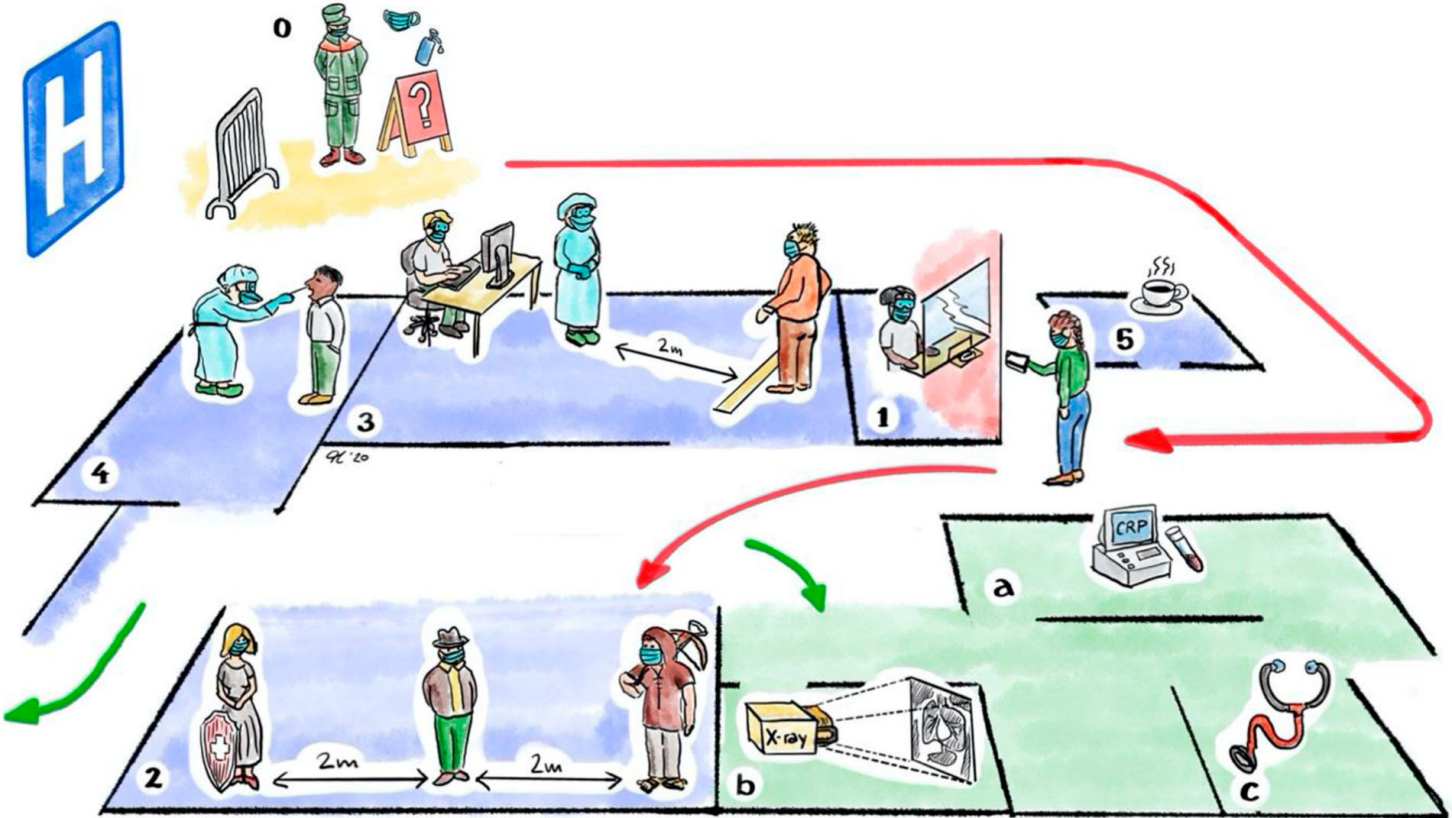
**(0–5):** Triage Unit set-up. **(A–C):** Triage *plus* set-up. **0**: Pre-Triage check point operated by civil protection. **1**: Patient reception, **2**: Waiting area, **3**: Clinical assessment room, **4**: Testing area for nasopharyngeal specimen collection, **5:** Staff room. **A**: Blood test, **B:** Chest imaging, **C:** Clinical examination.

I. *Patient Registration*
  When a patient is directed from a Pre-triage check-point to the triage unit, the first step is patient registration. At the patient reception area, the administration staff are located inside the pavilion while the patients queue outside, and a large glass visor is placed in between to avoid direct contact. At this stage, patients**’** data, including their mobile phone number and email address, are verified and recorded.
II. *Waiting Area*
  The second step is directing the patients to the large outdoor tent labelled as the waiting area. Because the weather in Switzerland, even in the springtime, can be a little cold [16] several heating lamps were installed. Our current data suggests that the average waiting time for each patient prior to clinical assessment was less than 10 min.
III. *Screening: History Taking & Assessment*
  The third step is collecting the patients from the waiting area and accompanying them into the main pavilion for clinical assessment by a physician. No patient is allowed to enter the building without wearing a surgical mask. For those individuals, especially small children, where the application of a face mask is not well tolerated, the assessment is performed outside. Prolonged stay (>15 min) inside the main pavilion is avoided. Again no physical contact is allowed and a minimum distance of 2 metres (≈3.28 feet) is maintained.
  Once inside the assessment room, the patient is asked a series of specific questions to identify if they display symptoms indicative of COVID-19, fulfill the criteria for a nasopharyngeal test, or require further investigations in Triage *plus*. A flow chart is designed to help facilitate the screening process for both adults and children (Appendices 1 & 2). Based on these flow charts, patients are either advised to self-isolate and discharged home *without* diagnostic testing, or undergo testing via a nasopharyngeal swab. In the latter group, individuals with no additional risk factors are then discharged home for self-isolation until the test results are obtained, or they are referred to Triage *plus* for further investigation. All patients who are discharged home are provided with information leaflets with appropriate advice and contact details according to their clinical scenario.
IV. *Testing*
  The fourth step is the nasopharyngeal specimen collection [17] itself, which is performed in a separate outdoor tent with good ventilation. Patients are provided with information leaflets which include details of the isolation protocol until further notification of their test results, which is usually within 24 h.
  If the patient is then discharged home, they leave the main Triage area using a separate exit. If, however, hospital admission is required, then the patient is accompanied by hospital staff to an isolated room inside the main hospital.
  Family physicians who have already screened suspected COVID-19 patients, but are unable to perform diagnostic testing at their practice, can take advantage of our Fast Track service. This involves a specially designed flow chart, which is completed by the family physician and handed to the patient directly. When this Fast Track document is presented at the reception area, the patient skips the evaluation process and goes directly to the testing area to receive the nasopharyngeal swab (Figure 2).
  Patients are notified of their results via an automated text messaging service if the test is negative or via a phone call by a trained HCW if the test is positive to provide detailed instructions for subsequent home quarantine. This service is coordinated directly by the Department of Infectious Disease and provides the opportunity for “telemedicine” or a remote clinical assessment via telephone [18,19] if necessary.

### Triage *plus*

If patients appear more severely affected and further medical investigations are indicated, they will be escorted to the Triage *plus* area. The Triage *plus area* is also located outside the main hospital building, and is directly adjacent to the main Triage pavilion. Here, patients are further assessed by a separate team of physicians who perform additional clinical examinations and investigations, including chest imaging and capillary blood C-reactive protein (CRP). Patients then leave Triage *plus* using the same exit as the other Triage patients unless they are admitted to an isolation zone within the hospital for further management.

## Observations

A total of 1265 patients were screened between 10 March 2020 and 12 April 2020 at our Triage unit. Weekdays saw more patients screened (average 40 cases/day) than weekends (average 19 cases/day) for a daily average of 33 screened patients. Of these, 112 (8.9%) tested positive for COVID-19. 73 (65%) of the positively-tested patients were female and 39 (35%) were male. The mean age for all patients was 43.8 years (SD 16.3 years). Distinguishing between genders, the mean age for females was 41.1 (SD 16.5) and the mean age for males was 48.6 (SD 14.9), with females being significantly younger than males (*p* < 0.001). Of note, 15 of the 112 (14%) positively-tested patients required hospitalization, including one patient who was transferred to ICU. The mean age of the hospitalized patients was 56.9 years (SD 12.9) with the majority (87%) being male. Following admission, there were 14 patients who were discharged home and one reported mortality (Table 1).

**Table 1.** Summary statistics for suspected COVID-19 patients who underwent testing in the triage unit.

	Gender	COVID-19 Positive (n)	(%)	Age (mean)	SD
**All Triage Patients**	Female	73	65	41.10	16.49
	Male	39	35	48.66	14.97
	*Total*	*112*	*100*	*43*.*74*	*16*.*32*
**Hospitalized Patients**	Female	13	86.7	59.00	11.31
	Male	2	13.3	56.61	13.59
	*Total*	*15*	*14*	*56*.*93*	*12*.*96*

Test to result time decreased from 48 h to a minimum of 4 h, as shortage of test reagents was solved and on-site testing was made available. According to recent literature, PCR sensitivity for nasopharyngeal specimen collection is approximately 67% (95%-CI 59-74) [20].

## Conclusions

The current COVID-19 pandemic is causing a paradigm shift for our globalized world in every sector. In any rapidly evolving crisis, certain principles remain key to combating a constantly evolving situation.

The design and implementation of the COVID-19 triage unit is based on principles, utilized by other shelter hospitals [21] and triage stations [22]. That is, rapid and simple construction, isolation from the main hospital, and readiness for a rapid scale-up whenever patient volume increases. Further advantages of having a triage unit outside the main hospital building include early screening and management of symptomatic patients, as well as prompt separation of patient groups and treatment pathways to mitigate nosocomial virus transmission.

The COVID-19 triage facility was set up within 72 h in the context of a national emergency and has to date, tested over 1200 individuals. The low percentage of positive cases and even lower percentage of those requiring hospitalization suggest, perhaps, that the Swiss population is adhering to the strict social-distancing measures imposed by the Swiss government. Data from our triage unit also indicates that younger women were more likely to be infected, but older men were more likely to be hospitalized. A conclusive rationale for this finding has yet to be elucidated.

There are of course limitations to consider. PCR analysis of nasopharyngeal swabs is only 67% sensitive. This suggests that up to one third of negatively tested individuals with clinical signs of COVID-19 were missed and allowed to potentially infect other healthy people. New rapid diagnostic tests, such as the Samba II recently developed by researchers at Cambridge University, can provide results in 90 min with a remarkable sensitivity of 98.7% [23]. However, these tests are expensive and are not yet available in Switzerland. Fortunately, our screening and testing strategy seems to have performed well so far, limiting the spread and speed of coronavirus dissemination and allowing upscaled hospital resources to cope with the influx of severe infections. This has certainly played a pivotal role in the remarkably low mortality rate reported in Switzerland despite the high number of diagnosed cases. Perhaps one of the greatest challenges we currently face is ensuring an efficient use of limited staff and resources combined with high-pressure time constraints.

Our triage unit has been able to successfully screen and control the spread of COVID-19 within our hospital premises, while at the same time continuing to perform emergency care without compromising patient safety. It is our hope that this article can provide some guidance and help other health care institutions develop and adapt effective triage strategies.
